# Phytohormones: plant switchers in developmental and growth stages in potato

**DOI:** 10.1186/s43141-021-00192-5

**Published:** 2021-06-17

**Authors:** Abbas Saidi, Zahra Hajibarat

**Affiliations:** Department of Plant Sciences and Biotechnology, Faculty of Life Sciences and Biotechnology, Shahid Beheshti University, Tehran, Iran

**Keywords:** Hormonal network, Development, Growth stages, Tuber formation, Potato

## Abstract

**Background:**

Potato is one of the most important food crops worldwide, contributing key nutrients to the human diet.

Plant hormones act as vital switchers in the regulation of various aspects of developmental and growth stages in potato. Due to the broad impacts of hormones on many developmental processes, their role in potato growth and developmental stages has been investigated.

**Main body of the abstract:**

This review presents a description of hormonal basic pathways, various interconnections between hormonal network and reciprocal relationships, and clarification of molecular events underlying potato growth. In the last decade, new findings have emerged regarding their function during sprout development, vegetative growth, tuber initiation, tuber development, and maturation in potato. Hormones can control the regulation of various aspects of growth and development in potato, either individually or in combination with other hormones. The molecular characterization of interplay between cytokinins (CKs), abscisic acid (ABA), and auxin and/or gibberellins (GAs) during tuber formation requires further undertaking. Recently, new evidences regarding the relative functions of hormones during various stages and an intricate network of several hormones controlling potato tuber formation are emerging. Although some aspects of their functions are widely covered, remarkable breaks in our knowledge and insights yet exist in the regulation of hormonal networks and their interactions during different stages of growth and various aspects of tuber formation.

**Short conclusion:**

The present review focuses on the relative roles of hormones during various developmental stages with a view to recognize their mechanisms of function in potato tuber development. For better insight, relevant evidences available on hormonal interaction during tuber development in other species are also described. We predict that the present review highlights some of the conceptual developments in the interplay of hormones and their associated downstream events influencing tuber formation.

## Background

Potato is one of the most economically important non-cereal food crops and an excellent stable food due to its high yield and great nutritive value. A potato tuber is considered a stem with a sprout and a number of axillary roots. The onset of sprout growth is observed following dormancy termination, involving several physiological and hormonal changes as well as incorporating intricate genetic regulatory networks [1]. Following sprout growth, tuber growth and development as well as tuber formation were regulated using a number of known phytohormones namely auxins, cytokinins (CKs), gibberellins (GAs), abscisic acid (ABA), ethylene, and strigolactones. Therefore, surveys of endogenous hormone levels and turnover are of special interest. Plant hormones are known to be markedly related to all stages of tuber development [2, 3].

The potato’s growth and developmental age can be divided into six major phases:

- Dormancy stage, freshly harvested tubers undergo a period of dormancy where visible bud growth is inhibited.
- In the tuber sprouting stage, transition from the dormant phase to the sprouting phase occurs where sprouts are developed from the eyes.
- The vegetative growth stage is initiated with the formation of sprouts until 8–12 leaves are formed. Also, the root system and the stolons are formed at this stage.
- Tuber induction and initiation begin with the emergence of tubers at the end of the stolons until the leaf system’s perfect development.
- Tuber development, at this stage a significant progress in tuber elongation and production occurs while the vegetative growth and the root systems cease growing.
- Tuber maturation, where the physiological aging of leaf structure and tuber skin’s tightening and thickening are initiated.

There are strong similarities between *Arabidopsis* and tomato in signaling pathways and the networks responsible for the control of potato growth and developmental stages, indicating conserved evolutionary processes across a wide spectrum of plants [4]. An evolutionary survey demonstrates that *Arabidopsis*, producing dry fruit, is the ancestor of tomato and potato producing fleshy fruits [5]. This is the reason for the commonalities of genetic control systems and regulatory mechanisms between the two types. Although information on the interaction between phytohormones at several levels of growth stages remains insufficient, with the appearance of omics approaches, important progress has been obtained in the identification of hormonal interplay. Previous research findings have led to the detection of overlapping patterns and hormonal pathways in the expression of genes related to hormone function across developmental stages in potato [6–9].

As in other plants, phytohormone synergy and their crosstalk can regulate tuber sprouting and developmental stages in combination with cell types, growth stages, and environmental conditions in potato, but the species-specific features should not be underestimated. For the last decades, some molecular and physiological surveys have revealed that the regulation of different aspects of tuber development is manifested through the individual hormonal pathways [4–10]. It is now clear that hormonal regulation includes consecutive levels in signal perception, transduction, transcriptional regulation, and complex metabolism exchange in the formation of potato tuber. Thus, the potato’s life cycle is reflected by the combined interaction of several phytohormones, whereas recent genetic evidence has revealed that hormones do not act alone in a linear pathway [11].

Some hormones possess synergistic and antagonistic roles in plant growth and developmental processes, acting as essential endogenous regulators in signaling pathways and subsequent responses [4]. In potato, for example, several hormones stimulate tuber sprouting and tuberization, whereas few hormones suppress them. For example, GA stimulated stem elongation in sprouting, vegetative stage of mother tuber, stolon initiation, and stolon development in potato [2–11]. Current evidence supports that CK and GA are required for bud break and tuber sprouting initiation, respectively [12, 13]. It has been reported that CK treatment stimulated bud break but not the sprout growth; however, another study has revealed that GA is a critical component in triggering further sprouting [13].

In addition to its role in bud germination, CK affects tuberization, resulting in an increase in the number of tuber formations. Although, the number of tubers increased under CK accumulation in stolon, however, tuber weight was reduced during tuberization processes. In addition to CK and GA, ABA is also involved in regulating tuber growth through the cessation of stolon apical growth in potato and the controlling of ABA/GA ratio [2, 14]. Also, ABA and ethylene suppress tuber sprouting [12]. Another survey showed that auxin is an essential player in the maintenance of seed dormancy. Auxin action in seed dormancy requires the ABA signaling pathway, emphasizing the critical roles of auxin and ABA in tuber dormancy [12].

Auxin plays an important role in potato tuberization, particularly in the processes of tuber initiation and growth. To understand signaling crosstalk among hormones, it is especially important to clarify the signaling mechanism of hormones in each stage of tuber development [2]. The basic functions and signaling pathways of the important hormones are briefly reviewed and a model is proposed to unravel the possible interplays among various hormones in terms of the dynamic maintenance of a normal plant [15]. Therefore, herein we have discussed the role of phytohormones in the context of their effects on diverse perspectives of growth and development and explain the pathways in which plant hormones regulate these responses, individually or in combination with other hormones. Particular emphasis has been placed on potato as it is an important model for molecular genetic analysis of tuber growth and development. Finally, we also highlighted the research context where more endeavors are required to improve our current insight on the role of plant hormones during tuber growth and development.

## Main text

### Transition from tuber dormancy to sprouting using hormone

After harvesting, potato tubers are dormant and do not germinate after planting. The length of the dormancy period depends on physiological and the relative concentrations of phytohormones. According to previous research reports, GA, CKs, and auxin are believed to regulate the termination of dormancy, while ethylene and ABA are needed to maintain bud dormancy in potato [16, 17]. It has been indicated that GA is sufficient to induce tuber sprouting. Hartmann et al. (2011) concluded that the onset of sprouting in potato influenced GA and CK [13].

It has been reported that the expression of CK oxidase/dehydrogenase 1 (CKX1) in transgenic potato plants led to lower CK content and did not respond to GA, while transgenic tubers harboring the *IPT* gene increased the endogenous CK content stimulated GA-mediated sprouting [12]. Hartmann et al. (2011) investigated the expression of GA 20-oxidase, which had slight effects on tuber sprouting and low modification in endogenous GA levels [13]. Besides GA and CK, auxin is one of the main growth-stimulating switchers influencing tuber dormancy and sprout development [18]. The transition from dormancy to seed growth is facilitated by a coordinating network of auxin and ABA signaling in complex physiological processes [19]. Previous findings have indicated that auxin and ABA regulate seed dormancy, synergistically [19, 20]. Auxin plays a vital role in inducing and maintaining seed dormancy. Recent studies have demonstrated that ABI5, a basic leucine zipper (bZIP) transcription factor, controlled seed dormancy by ABA and auxin hormones [10–21].

Auxin regulates seed germination through AUXIN RESPONSE FACTOR (ARF), whereas ABA inhibits seed germination through ABIs. Therefore, there is a molecular link between ABA and auxin [22]. Liu et al. (2012) suggested that *ARF10* and *ARF16* genes are activators of ABI3 transcription [19]. These results revealed that *ARF10* and *ARF16* are needed to preserve ABI3 expression. The *arf16arf18* double mutants significantly reduced seed dormancy as compared with the wild type. In addition, [22] indicated that mutation in the *ABI3* gene inhibited the effects of auxin and ABA on seed germination in *Arabidopsis*. A recent survey showed that ABI3, ABI4, and ABI5 are core determinant factors in the transition from dormancy to sprouting [21–23]. These TFs induced the downstream target genes regulating seed germination. In potato, *StABI5* plays an important role in the regulation of tuber dormancy through the controlling of the auxin signaling pathway. In addition, *StABI5* regulates the genes (i.e., SUAR and AUX) downstream of the auxin signaling [24].

An experimental study in potato has revealed that auxin concentration is at its highest at tuber dormancy and begin reducing during bud growth [18]. Auxin is the main cause of differentiation processes in buds and serves as a bud growth stimulant. Another survey has shown that there is a positive correlation between auxin concentration and termination of tuber dormancy [18–25]. Faivre-Rampant et al. [26] suggested that the *ARF* gene can be considered a marker for meristem reactivation in potato tubers. In potato, *ARF1* gene expression was significantly enhanced after tuber dormancy breaking, probably related to tuber dormancy and sprouting [19]. The relative expression level was modified in different tissues such as stem, root, leaf, shoot, and tuber. *ARF1* gene expression was downregulated in dormant tubers, whereas it was upregulated in the sprouting tubers [22].

In addition to CK, GA, ABA, auxin, strigolactones (SL) had strong inhibitory effects on tuber bud growth. Another report concluded that SL does not influence bud growth alone [27]. A study revealed that SL affects GA and CK actions to inhibit potato buds [28]. Experimental research has indicated an additional role for auxin together with SL in the inhibition of bud outgrowth and the formation of shoot and root architecture [29].

### Role of hormone in tuber sprouting

Tuber sprouting demonstrates the first step of tuber development when potato tubers pass through the dormancy phases. With the initiation of sprouting, hormonal and metabolic changes as well as the level of gene expression in tubers are accompanied. Additionally, tubers become a source organ for the growing and developing sprouts [13]. Although many researchers have investigated the molecular changes during sprout growth, the molecular mechanisms and hormonal interplay triggering tuber sprouting are yet ambiguous [12].

Previous surveys at the molecular and hormonal levels have established a mediating role for a SPINDLY (SPY) in the interplay between GA and CK during plant development [30, 31]. Qin et al. [2011] revealed that SPY acts both as an inhibitor of GA response and a positive regulator of CK response in *Arabidopsis*, suggesting a model for SPY as a main modulator of the interplay between both GA/CK signaling pathways [30]. In *Arabidopsis* and petunia, *spy* mutation suppresses the impacts of GA deficiency on germination, and overexpression of SPY prevents seed germination.

**Fig. 1:**
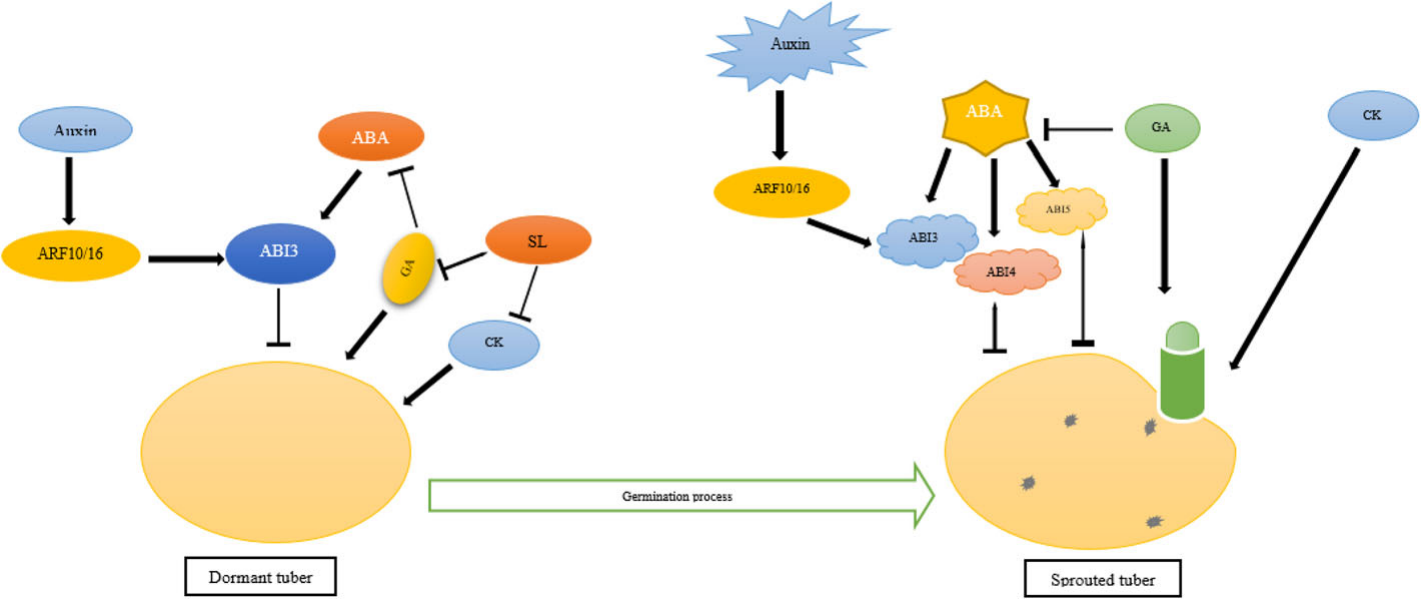
A network of reciprocal interaction of some hormones in transition from dormant to germinated seed. Transition from dormancy to germination of seed are two separate but continuous phases. Freshly matured seeds are dormant and include a high level of ABA, auxin, and SL, and a low level of GA and CK. After seed dormancy is broken, non-dormant tubers begin germination (right side of the figure). Different hormones influence this process by controlling the ABA/GA and auxin/GA balance at the signaling levels. Transcription factors ARFs, ABI3, ABI4, and ABI5, as signaling negative regulators, play key role in this process. The initiation of sprouting in potato influenced GA and CK. ARF10/16 and ABI3/4/5 are involved in the interaction between ABA and auxin, having a potential role in tuber dormancy maintenance. Arrows and T-bars show positive and inhibitory effects, respectively

Crosstalk between GA/CK occurs at various stages of plant growth and development in *Arabidopsis*. In *Arabidopsis*, it has been reported that auxin is considered one of the GA-response signaling components during germination [32, 33]. A previous survey has revealed that CKs seem to stimulate either leafy shoot or tuber initiation in potato. Lately, Muthoni et al. [2014] demonstrated that the synergy of CK, auxin, and GA causes the onset of growth and morphogenesis as well as the elimination of blocking the G1/S phase in potato bud sprouting [17]. Earlier studies have revealed that acetylation of histone proteins cause termination of tuber dormancy in potato [34]. Evidence also suggests that ABA and auxin play a synergistic role in sprouting, but auxin, GA, and CK are responsible for fine-tuning the core cell cycle in cell division (Fig. 1).

A current survey has demonstrated that the synergism observed among three hormones, namely GAs, CK, and auxin, play a key role in the control of sprout development [35]. Individually, any of these hormones can only stimulate bud break but not the sprout growth, whereas their combined actions can induce the initiation of tuber sprouting [16]. For example, it is concluded that CK alone can help to overcome ABA-inhibited germination in lettuce. However, most of the published literature has demonstrated that CK is implicated in a wide interplay between GA and auxin in the regulation of dormancy and germination [34]. Auxin and CK act synergistically and antagonistically to control cell division and bud formation in tomato, respectively. These findings show the involvement of multiple phytohormones in the regulation of tuber sprouting and suggest that there is an accurate balance between their metabolism and responses in sprouting. This implicates that the coordinated function of auxin and/or GA and/or CK through their metabolism and/or signaling regulates the activation of cell cycle genes during dormancy and tuber sprouting stages.

### Cellular bases of dormancy and sprouting

Dormancy is defined by the absence of visible growth whereas molecular events are active in the expression of proteins involved in the cell growth of dormant meristems. Hartmann et al. [13] revealed that re-activation of meristematic activity precedes tuber bud growth and is accompanied by enhanced cell division, causing tuber sprouting. The content and activities of proteins involved in the cell cycle and division are controlled by the coordinate synthesis and action of a number of cyclin-dependent kinases and their downstream targets. Cyclin-dependent kinase (CDK) activated by D-type cyclins will transfer cells from the G1 into the S phase [36]. At the cellular level, dormancy likely occurs by a G1-phase arrest of the meristematic cells. Escape from this arrest needs D-type cyclins (CycD) which involves complex processes to transmit from the G_1_ to the S phase. There are documents indicating that CK induces the expression of genes coding for promoting the termination of tuber dormancy, namely CDKA and its targets [36, 37].

CK stimulates cell division in plant tissues depleted of hormones, a causal effect of G-1 cell cycle block. In addition, application of CK can lead to the G1/S transition and divide the cycling cells in meristem by inducing G0 cells to enter the cell cycle [38, 39]. However, growth inhibition in dormant tuber meristems is a consequence of the arrest of bud meristem cells in the G-1 phase of the cell cycle [40]. Exogenous application of CK stimulates termination of tuber dormancy, and endogenous levels of CK can cause the onset of sprout growth. Numerous studies have revealed that zeatin treatment leads to the upregulation of CycD3 in *Arabidopsis* and *Camellia* [41–43].

During the transition from the dormancy to the sprouting phase, the expression of genes encoding histones H3, H4, H2B, and other proteins namely map kinase, gamma tubulin, and ovule/fiber elongation protein are implicated in cell division and the initiation of sprouting [44]. Different transition points G1-S, G2-M, and M-G1 phases were regulated by CDK (checkpoint of cyclin-dependent protein kinase), CDK binding to a D-cyclin to initiate cell cycle. Campbell et al. (1996) showed that D-cyclins regulated Cdc 2 kinase [40]. These proteins are necessary for G1 to S control. CK-induced cyclins can react with CDK to initiate the G1 to S phase. A previous study revealed that GA may influence the Cdc2 kinase level at the G2-M checkpoint of the cell cycle, and may also enhance the rate at which cells are produced [39]. CK has also been found to be active in the G2-M transition of the cell cycle where induction of a histone-H-kinase, cdc2, takes place [45]. Also, it has been suggested that CK may be implicated in the disruption of growth inhibitors, allowing an opportunity for GA to function [46, 47].

Aside from CK and GA, auxin mostly accumulates in the meristem and bud primordia in dormant tubers. At the end of storage buds, auxin was identified only in lateral bud primordia from growing buds. A negative correlation was reported between auxin content in buds and the end of dormancy [18]. It has been reported that the expression of protein kinase StCDPK1, as a probable activator of the auxin transporter StPIN4, was observed in the vascular tissues in dormant tubers, whereas upon tuber sprouting, its expression enhanced in buds and young shoots [48]. The proposed model suggests that the regulation of StCDPK1 expression varied in specific tissues using miR390 at the post-transcriptional level [49]. In several studies, auxin signaling regulated the cell cycle directly or through crosstalk with other plant hormones [18–49]. Few reports have revealed no direct role for auxin in tuber dormancy [3–26], whereas another report has concluded that low auxin concentration promoted sprout growth after dormancy had terminated in potato [17]. Another survey showed that auxin inhibits the production of CK, a plant hormone that is needed to induce both CYCD3 and CDK3 expression [50]. Further, another study revealed that the interaction of auxin and CK controlled cyclin D3 in the cell cycle during tuber dormancy [13].

### Role of hormones in vegetative growth of mother tuber

Potato is propagated by vegetative methods and its tubers possess eyes and nodes. The plant produces underground shoots known as stolons, which grow along the ground surface [51]. At the vegetative growth stage, the plant is supplied with starch and carbohydrate stored in the mother tuber. Later, leaves develop, the process of photosynthesis begins, and the plant can become capable of nourishing itself in preparation for new tuber growth [52]. Vegetative growth is extremely regulated by phytohormones [53]. Hormones are essential for plant growth and development namely structure of the plant, seed growth, flowering time, senescence of leaves and tubers, and some other processes [54].

### Shoot and root development

Based on a recent study, GA_3_ was found to promote vegetative growth, namely elongation of stems and expansion of leaves [55]. It has been reported that GA_3_ could be elevated on internode elongation and shoot growth [56]. Aside from its effects on dormancy termination, GA_3_ influences vegetative growth, yield, and tuber quality [55]. A previous study has shown the involvement of auxin-GA crosstalk in regulating various cellular responses, namely GA biosynthesis and signaling in root growth and development [57]. GA destabilizes DELLA proteins such as RGA and GAI, functioning as growth repressors. Root development depends on the mechanism of GA action. Also, cell expansion is regulated using the DELLA protein. GA promotes root growth by targeting DELLA degradation in each one of these elongation zone tissues. Conjugation of GA to GID1, its soluble receptor, causes an increase in the interaction of GID1-GA and DELLA proteins, resulting in a change in their ubiquitin-proteasome path [58]. The DELLA protein, a key negative regulator, is essential for GA function [59]. GA-GID1-DELLA complexity changes in the presence and/or absence of GA. In the absence of GA, DELLA conjugates to protein complex and inhibits TFs and when GA is present, GID1 triggers the disruption of DELLA and the TFs [60].

Reduction of auxin transport delays GA-induced disappearance of RGA from root cell representing requisiteness of auxins in GA-regulated control of root development [61]. A recent survey demonstrated that GA-auxin interaction modulates root development [62]. GA mutant deficient in both synthesis and signaling induces an increase in root formation [63, 64]. It has been reported that auxin amount, like the *PIN9* gene has been increased both in GA-deficient and GA-insensitive mutants of Populus root. These results indicated that in the mutants (GA-deficient and insensitive), auxin contributes more significantly to root formation as compared to WT, confirming GA-auxin interplay in the root development. The impact of auxin on GA biosynthesis can be related to the transport of auxin by AtPIN1 resulting in the disruption of AUX/IAA and activation of ARF7 TFs. There is another report that auxin regulates root formation via ARF7 and ARF19 [65].

Besides GA_3_, auxin is also known to induce stem elongation [66] and root development [65, 67]. Also, auxin is synthesized in meristems and young tissues and transported through the stem to sink tissues [68]. Auxin induces expansion of stem or coleoptile parts, commonly related to enhanced water uptake. Also, auxin strongly inhibits root growth at concentrations promoting stem expansion. Based on another study, the extension of the cereal leaf is stimulated by both auxin and GA [69].

Auxin biosynthesis and its transport in cells are largely influenced by the processes of plant development [70]. Auxin transport is performed using the PIN family. Upregulation of the expression of two *PIN* genes was observed at different stages of potato tuber formation. StPIN1 and StPIN4 were expressed in all plant tissues including young tubers, whereas StPIN2 and StPIN5 were active in stolons and roots, respectively [3]. A previous study revealed that upregulation of two *PIN* family genes causes an auxin content increase in the stolons [3].

The AUX1, one of the most important AUX/LAX protein families, imports auxin into the cell. Mutation of the corresponding gene impaired gravitropism and altered growth of lateral roots and shoots. To date, *AUX* genes in the *Solanaceae* family have not been surveyed adequately, data being only available in tomato. However, due to the high conservation of auxin signaling, auxin transport may be assumed in a similar way in the closely related potato and tomato plants. For example, *SlLAX1-3* genes were expressed in the flower, fruit, and other parts of the plant, whereas other genes were expressed in vegetative organs [71]. Another class of transporter proteins includes members of the ATP-binding cassette transporter (ABCB) family, participating in auxin transport across the hypocotyl and root.

Auxin movement in roots is controlled through AUX1, ABCB19, PIN1, PIN3, and PIN7 at lateral root initiation and elongation stages. Also, the movement of auxin from the shoot to the root requires AUX1, PIN2, and ABCB4, mediating this polarity of auxin transport. Mutation of these genes revealed altered auxin in *Arabidopsis* [72]. The structure of auxin signaling and transporter are highly conserved, making them an ancestral-like auxin transport in the cell. One of the first studies of *ABCD* genes was performed on potato. These genes are homolog of MDR. ABCB1/19, a member of IAA transporters, is active in stolon tips [73].

Auxin signaling is regulated by ARF transcription factors, *ARF1* and *ARF19*, controlling root formation in *Arabidopsis* by regulating the expression of LATERAL ORGAN BOUNDARIES DOMAIN 16/ASYMMETRIC LEAVES2-LIKE 18 (LBD16/ASL18) and LBD29/ASL16 [62]. It has been reported that *ARF17*, *ARF6*, and *ARF8* were positive regulators of the auxin-inducible genes (i.e., *GH3.3* and *GH3.5*) for fine-tuning of root initiation in *Arabidopsis* [74]. In *Arabidopsis*, overexpression of *ARF8* causes short hypocotyls and repression of lateral root formation [75]. In addition, overexpression of *SIARF6A* enhanced the photosynthetic rate and accumulation of starch and sugars, while knockdown of *SIARF6A* resulted in undesirable phenotypes in tomato leaves and fruits [76]. In rice, downregulation of *OsARF1* leads to short leaves and growth retardation. These results suggested that *OsARF1* has a vital role in vegetative organ and seed development [77]. In potato, overexpression of *AtYUC5* homolog, represented by high auxin, causes narrow downward leaves, enhanced height, and erect plant stature [78]. Of the phytohormones surveyed, GA exerts the largest effect on vegetative growth. Exogenous application of GA enhanced plant height and whole leaf area in potato [78].

**Fig. 2:**
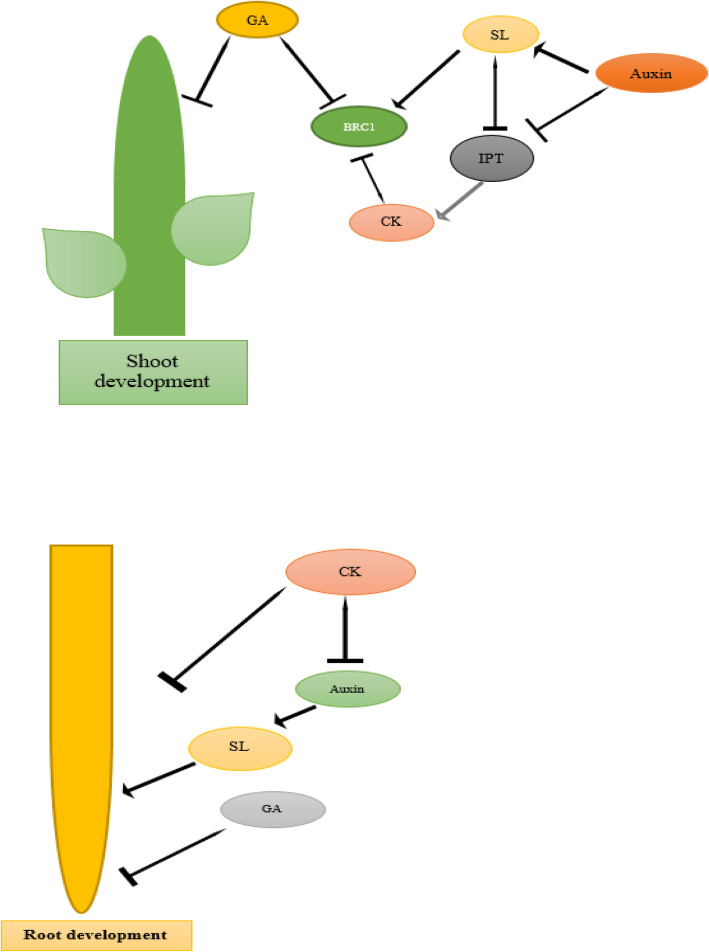
Model of the interaction among auxin, CK, SL, and GA in the control of shoot and root formation. **A** Auxin, cytokinins (CK), and strigolactones (SL) are implicated in the hormonal regulation of *BRC1* expression in shoot branching. In this regulation network, Auxin, GA, and SL are suppressors and CK is a stimulator of bud growth. **B** GA and CK are inhibitors and auxin and SL are activators of root development

GA is involved in stem cell growth, leaf, and other aerial sections by causing cell enlargement and enhancing intermodal longitude, whereas auxin stimulates cell enlargement and elongation, especially during the initiation of shoots and roots [9]. The evidence indicates that GA and auxin biosynthesis and transport mostly promote leaf growth and expansion, internode length, shoot growth, and root elongation. Despite their clear roles in tuber growth and development, only few reports on their role during vegetative growth are available. Thus, one of the major challenges for future work remains the complete insight of the molecular mechanisms underlying vegetative growth and the interactions among hormones, as it is anticipated that events taking place at this stage might be of critical importance at tuberization (Fig. 2).

Besides GA and auxin, CK controls cell division and elongation in the root zone. A previous survey has shown that CK and auxin have antagonist functions in root development. Application of exogenous auxin increases root growth, while CK reduces it [79, 80]. Street et al. (2016) concluded that the CK signaling pathway incorporates histidine kinase receptors (AHKs), histidine-containing phosphotransfer proteins (AHPs), and response regulators (ARRs) [80]. Signaling is started by CK binding to and inducing autophosphorylation of the AHK. Receptor phosphorylation was accomplished in AHP and ARRs, ARRs playing a key role in CK signaling [81].

Lomin et al. (2018) indicated that the *StHK3* gene is expressed in leaves and stems, whereas the expression of StHK3/4 levels was relatively high in roots [81]. A previous survey suggests that targets of ARR1 contain the auxin-response repressor gene (*SHY2*). SHY2, a repressor protein and a member of the AUX/IAA family, is expressed in the root tissues and is induced by CK. CK was revealed to directly activate transcription of auxin repressor IAA/SHY2 (SHORT HYPOCOTYL2) through the AHK3 receptor and ARR1 and ARR12 response regulators. This phenomenon led to the attenuation of auxin reactions and decreased expression of PIN transporters [82]. As a result, a reduced abundance of PINs restricts the auxin source to the root, thereby limiting its growth at this region [81]. Besides this transcription-based regulation of auxin activity and dissemination, CK was likewise found to adjust the endocytic transporting of PIN1 by resending this membrane protein for lytic depression in the vacuoles [82]. Lately, Marhavý et al. [83] provided more evidence for CK activity in the peripheral region and symmetry of lateral organ initiation. CK biosynthesis and breakdown is controlled by *IPT* and *CKX* genes, respectively. CK action is based on an AHK signaling pathway that is an initiator of phosphorylation cascades and later as an activator of AHP and ARR.

Analysis of AHP6 (*Arabidopsis* HISTIDINE PHOSPHOTRANSFER PROTEIN 6) expression pathways combined with monitoring of auxin and CK susceptible reporters indicates that AHP6 acts as a repressor of CK signaling. AHP6 controls expression patterns of CK in the shoot and root. The CK may inhibit auxin function using AHP6 through CK feedback on the PIN transporters [84]. A previous study has revealed that auxin stimulates the transcription of AHP6, acting as a negative regulator (repressor) of CK signaling [82–86]. There is strong evidence that AHP6 is regulated directly by auxin.

Shoot branching is controlled by auxin, CK, and SL, known as managers of bud growth. Although ABA and GA regulate part of bud growth, however, their roles have been less reviewed as compared with other hormones (auxin, CK, and SL). The impact of GA on bud growth differs strongly among species. GA inhibits shoot branching in tomato and rice [87]. The accurate mechanism behind their impact stays ambiguous and might be linked to the change of SL biosynthesis and sugar sink [88, 89]. Application of exogenous ABA inhibits bud growth, where bud ABA content is negatively correlated to the bud’s ability to grow out. It has been reported that ABA acts downstream of auxin signaling (AUXIN-RESISTANT 1 AXR1), MORE AXILLARY BRANCHED (MAX) signaling (MAX2), and BRANCHED1 (*BRC1*) gene [90]. AtBRC1 induces ABA synthesis by upregulation of NCED3 expression, encoding a key ABA-synthesis enzyme [90].

Application of synthetic SL GR24 to the *rms1* mutant plant retarded lateral bud growth, representing an involvement in branch formation inhibition [63]. Exogenous application of SL inhibited shoot branching and bud growth [91]. The co-ordinated regulation of plant growth needs effective communication among hormones. A good example of the interaction of CK, auxin, and SL is in shoot branching, where CK promotes growth while auxin and SL play the role of bud growth repressors. CK acts antagonistically with SL. There is a hypothesis that CK and SL act as long-distance secondary messengers for auxin, but do not require auxin to act. An experimental study demonstrated that auxin moves in the stem and inhibits CK levels as well as promotes the expression of SL biosynthesis genes. These hormones can regulate budding and branching with *BRC1* mediating these processes. *BRC1* encodes TF needed for branching inhibition [27]. Another study revealed that CK can inhibit *BRC1* expression and stimulates bud outgrowth [92].

Auxin cannot directly control *BRC1* expression because there is not an adequate amount of auxin to transport the *BRC1* from the stem to the buds. Auxin can indirectly stimulate *BRC1* expression in buds through CK and SL [71]. Auxin indirectly inhibits bud growth by reducing CK levels, where CK contents could determine bud growth. Another study showed that high CK levels in buds lead to the activation of buds through downregulation of *BRC1* expression [93]. Based on another study, auxin inhibits the expression of *isopentenyltransferase* (*IPT*) gene in the stem and as a result downregulating CK levels in the xylem [94]. According to another study, *BRC1* is repressed by the regulation of ABA, playing important roles in the plant life cycle [95]. There is evidence for the ABA role in regulating bud growth, exhibiting a positive correlation between a reduction in the ABA content and break of bud dormancy [95].

Besides auxin, CK, and SL, gibberellin (GA) might also be implicated in the regulation of *BRC1* expression, even if the mechanisms are yet unknown. In rosa species, GA highly increases bud growth, whereas in woody plants, GA and CK promote lateral bud growth but negatively affect BRC1/2 expression [96].

### Role of hormones on tuber initiation and induction

Tuber initiation is commonly preceded by stolon initiation in potato. During stolon development, cell divisions are limited mainly to the apical bud and subapical region. Stolon development ceases after tuber initiation [97]. During tuber initiation, changes in the area of cell division and cell enlargement in the stolon will give rise to the young tubers through swelling [3]. Tuber formation is associated with a cessation of stolon growth, a phenomenon known to be controlled by hormones and endogenous signals. Tuber induction and formation involve leaf-triggered mobile signals, florigenic, and tuberigenic signals [98]. The mobile signal flowering locus T (FT), the main component transported from the leaf to both the apex and the stolon, is involved in the initiation of particular developmental processes namely flowering and tuberization [99]. The potato *FT* homologs, StSP6A and StSP3D, induce tuberization, whereas StSP5G, as a repressor of StSP6A transcription in leaves, is involved in the inhibition of tuberization [100]. It has been observed that StSP6A and GAs, as mobile molecules, regulate tuberization [98]. In potato, ABA, CK, auxin, and jasmonic acid stimulate tuberization, whereas GA has a suppressive role [101]. In addition, the process of tuber initiation has been surveyed in relation to levels of plant hormones namely CKs, GAs, auxins, and abscisic acid [11–104]. A previous study revealed that the JA is involved in the growth and induction of tuber formation, reduction of leaf primordia length, enlargement of meristems, and increase in cell expansion in potato [101].

The endogenous level of GA inhibits tuber formation and promotes stolon elongation [104]. The inhibition effects of GA on potato tuberization have been identified; however, ABA acts as a regulator, reducing the GA level and increasing levels of hormones required for tuberization [104]. The evaluation of endogenous ABA revealed an increase of ABA levels under tuber-inducing conditions and a decrease under tuber formation. As of yet, the ABA function in tuber formation is not crystal clear and has been proposed that it stimulates tuberization through suppression of GA inhibitory effects [105].

Another study revealed that GAs are the most likely candidates for inhibition of tuber formation [106]. Bamberg and Hanneman (1991) suggested that mutation in GA genes leads to tuberization induction [107]. Besides GA, CK influences growth and developmental processes namely, shoot and root elongation, shoot regeneration, and meristematic activity. Many recent surveys have revealed development-dependent reciprocal interactions between the two hormones, where CK prevents the production of GA and elevates its deactivation and conversely GA prevents CK responses [2–47]. Reciprocal interplays between GA and CK were regulated using two players controlling the balance between GA and CK.

KNOX proteins, as the first player administering the balance between the two hormones in the SAM, produce CK through two pathways, directly inhibiting GA synthesis and indirectly elevating GA deactivation. SPY, as the second player, regulates the balance between the two hormones via the suppression of GA signal and the increase of CK responses, *SPINDLY* (*SPY*) has a major impact on the GA signaling pathway [101]. SPY is required for DELLA activity, causing an elevation of the CK signaling pathway. It is suggested that the *spy* mutant can suppress the inhibition of root growth by CKs [29]. Analysis of microarray data from seed showed that CK inhibits expression of genes encoding GA biosynthesis enzymes GA20ox and GA30ox, whereas promotes expression of GAI and RGA [32].

Molecular experiments have revealed that the potato proteins (i.e., POTH1, KNOX) negatively control GA levels. POTH1 is involved in vegetative growth, accompanied by a reduction in GA levels. Reduction of GA20 oxidase levels was observed through conjugation to particular elements of POTH1 in regulatory sections of the *GA20 oxidase* gene to inhibit its function. GA20 oxidase, a vital enzyme in the GA biosynthesis pathway, is essential for the production of inactive GA20, the precursor of active GA1. GA20 oxidase encodes functionally identical enzymes with various patterns of tissue-specific expression. For example, one of the *GA20 oxidase* genes, known as *StGA20ox1*, is highly expressed in the shoot and leaf, but is expressed at low levels in stems, stolons, and tubers. *StGA20ox2* is relatively in higher levels in stolons and tubers while it is at relatively low levels in fruits and developing seeds*. StGA20ox3* accumulates in stems, roots, stolons, and tubers, nonetheless at a lower level than the other two genes [108].

Earlier surveys at the molecular and genetic levels have established a role for POTH1 and StBEL5 in mediating the interplay between GA and CK [109, 110]. Also, POHI1 and StBEL5 affected tuber growth by decreasing GA levels and inducing cell growth with enhanced CKs at the stolon tip [94]. Overexpression of POTH1 promotes an increase in tuber number, whereas overexpression of StBEL5 increases tuber formation and tuberization [110]. In addition, it has been reported that overexpression of POTHI and StBEL5 elevates CK levels in potato transgenic lines [110].

Besides GA and CK, auxin is also involved in inducing tuber initiation in potato. Auxins are implicated in the process of cell enlargement and have been shown to have a key role in flower development and lateral root formation. In addition, auxin plays a critical role in tuber initiation and development. During the tuber initiation stage, changes in the plane of the cell division occur in the stolon region, giving rise to the young tubers through swelling [111]. The endogenous auxin level is positively correlated with the tuber formation particularly in the stimulation of cell division and differentiation. Genes involved in biosynthesis (*YUCCA*), auxin response factor (*ARF*), and transport (*PIN* gene family) are differentially expressed during tuber formation [3]. Downregulation of the *StYUCCA19061* gene expression during the tuber development stage indicates its potential function in the onset of tuber initiation.

In addition to auxin, significant changes at the tuber initiation stage occur with gibberellin signaling. The gibberellin content in stolons decreases sharply, partly due to the decrease in gibberellin flux from the leaves and partly due to the processes in the stolons themselves.

Since cell division and elongation are the initial events prior to tuber initiation and induction, CKs should be present to trigger tuberization [112]. This does not mean, though, that a modification in the plant CKs level is the phenomenon that triggers tuberization. Another survey demonstrated that CKs increase in shoots as plants are stimulated to tuberize, but the increase continues relatively slowly until tuber has been initiated [2]. Experimental investigations indicated that CK was accommodated into stolon ends prior to tuber formation. Exogenous application of CK and overexpression of *IPT* gene cause reduction of tuber weight. These results suggest that CK may be responsible for triggering stolon branching rather than tuber induction [113].

Enhanced endogenous CK caused cell division of shoot outgrowth through the protein kinase activity of CDK/CYCLIN in potato, whereas application of exogenous CK caused an increase in CDK activity [36]. Although CK promotes leaf development and shoot growth, it inhibits root growth and development in potato [113]. In potato, exogenous CK may convert a stolon into a leaf-bearing shoot. However, Harmey et al. [114] observed that auxin application induced bigger tubers at an earlier stage. Auxin content was high at the tuber initiation stage and low at the tuber development stage. Experimental studies show that tuberization occurs most effectively at a certain concentration close to the naturally occurring auxin/CK concentration ratio [115]. A positive correlation has been established between the cell multiplication rate and the concentrations of auxin and CK at the initial tuber growth stage [116]. An intricate network of auxin and CK interplays has been observed in the activity of root and morphogenesis. CK regulates the auxin pathway by influencing the expression of its signaling components.

**Fig. 3:**
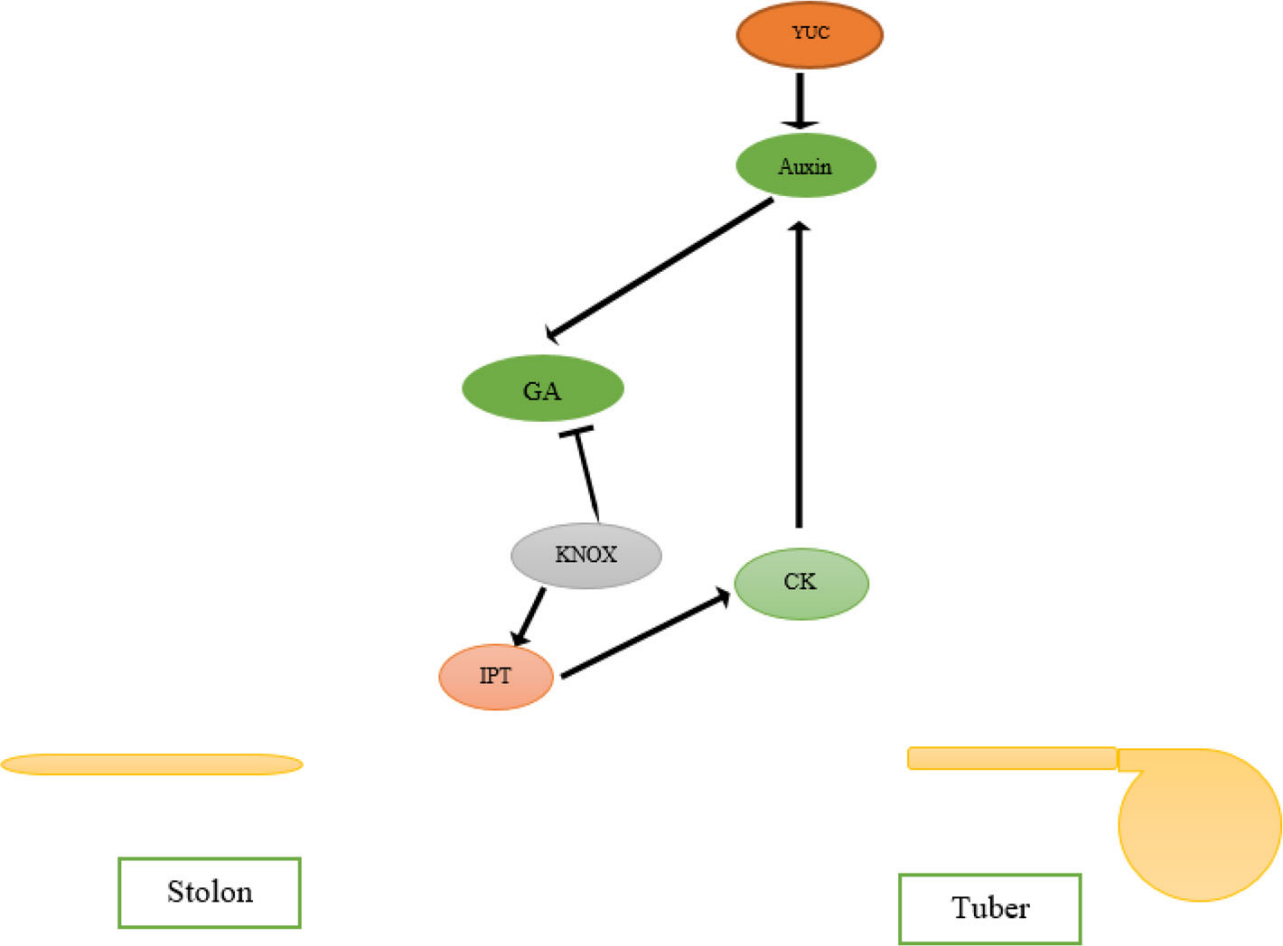
An overview of the interaction between auxin, GA, and CK hormones during tuber initiation. CK regulates the auxin pathway by influencing the expression of its signaling components. Auxin decreased significant levels of GA at the tuber initiation stage with gibberellin signaling. KNOX proteins promote activation of CK and repression of GA biosynthesis and signaling. IPT is the enzyme responsible for the rate-limiting step of cytokinin biosynthesis

Further, CKs play an important function in the regulation of tuberization in potato through cell division and proliferation. Actually, CKs are an appropriate source for tuber initiation. However, overexpression of the CK oxidase inactivation enzyme results in a reduced number of tubers per plant. In addition, overexpression of the *IPT* gene results in tuber yield reduction. Consequently, it is difficult to assign a particular role to CK in tuberization [13]. Overall, the tuber initiation phase is established by multiple independent physiological aspects. It is considered that more investigations are needed to determine the precise mechanisms of involved hormones, to delineate the interconnection and effects between hormones, and detection of other roles that hormones play in tuber initiation (Fig. 3).

Perception of the photoperiodic signals in leaves takes place through photosensitive proteins namely, photochromic B, blue light receptor, circadian proteins, the sucrose transporter SUT4, and several other proteins [2–106]. These primary receptors of tuberization transport the signals of a particular set of genes encoding regulatory proteins (StCO, StCDF, StBEL5, POTH1) and miRNAs. Different forms of polypeptides and/or bind/miRNA are moved towards stolons [2]. This movement is simplified by the carrier proteins StPTB1 and StPTB6 [97]. At this time, inhibition of *StCO* gene activities in the leaf causes the expression of the *StSP6A* gene. The *StSP6A* gene encodes a paralog of the FT (Flowering Locus T) protein, called florigen [117].

The StSP6A protein is translocated from leaves to stolons and is specifically important for the transition of stolons from the elongation stage to the tuberization stage [118]. An earlier survey has presented evidence for GA and CKs acting as an inhibitor and promoter in tuberization, respectively [119]. In addition, the interaction of CK and auxin guarantees tuber development. In soybean, the interaction of auxin-CK is important in the soybean callus bioassay when both hormones are needed for cell division and expansion [120]. There is evidence that individual tuberization processes themselves may be under particular hormonal control [2]. However, tuber growth and development are influenced by other environmental conditions, namely daylength, photoperiodic signaling, and cellular and transcriptional mechanisms [94]. Therefore, we surveyed in detail the available literature on the relative roles of these hormones, individually and/or in combination, on different factors of tuberization.

### Tuber development controls by GA, CK, and auxin hormones

Tuber growth is when a stolon starts to distinguish and swell, to produce the tuber [121]. Upon initiation of tuberization on a longitudinal stolon, it is well established that both cell division and expansion are required for tuber growth and development. Some describe that cell division precedes cell enlargement [122, 123]. During the enlargement stage, tuber cells extend with the accumulation of carbohydrates, nutrients, and water. Tubers are considered a major sink for carbohydrates and an inorganic nutrient storage [124]. Tuber growth and development occur at the time of least leaf area production and decreasing leaves. Tuber initiation occurs when the plant is at its maximum leaf area stage.

A study has revealed that the higher the leaf area, the higher the tuber growth [125]. Also, other studies have indicated evidences for phytohormones acting as chemical switchers, namely GA as inhibitor and CKs and auxin as promoters of tuber development in potato [113–126]. The expression of *StGA20ox1*, implicated in GA degradation, is induced prior to stolon swelling [108]. In agreement with our findings, overexpression of *StGA20ox1* (active GA biosynthesis gene) delayed tuber development in potato [3].

During stem growth as well as tuber initiation, GA stimulates cell elongation and expansion in stolons [2]. However, GA plays an inhibitory role in tuber growth and development. Analysis of the endogenous GA content has revealed that after tuber development, GA levels in swelling stolon tips are reduced [127]. BEL5 is a transcription factor interacting with the POTH1 to initiate tuber development. *BEL5* RNA is relocated from the leaves to the stolon tips to induce tuber formation [110]. Besides the GA, CK plays a prominent role during tuber formation namely tuber initiation, tuber setting, and enlargement [128]. In addition, CKs increase cell divisions in *Arabidopsis*, tomato, and tobacco and are implicated in cell proliferation in the early stages of tuber growth [121].

**Fig. 4:**
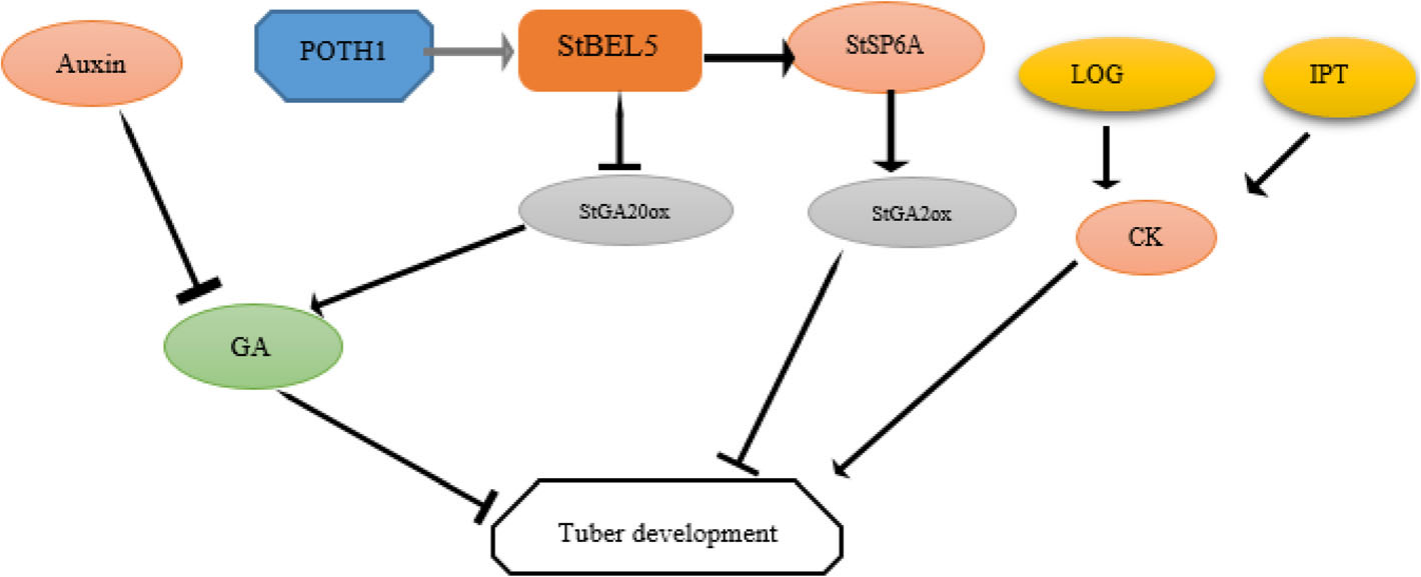
The regulatory network of the involved players during the onset of tuber development in potato. StSP6A protein and *StBEL5* mRNA are the molecular signals in tuber development. POTH1 is a negative regulator in GA signaling during the tuberization stage. GA acts as a tuberization repressor and StSP6A and StBEL5 play roles as tuberization promoters. Auxin and CK promote tuber development. StSP6A protein and StBEL5 RNA are considered long-distance tuberization signals, moving through the phloem from the leaf to the stolon. *StGA20ox* delayed tuber development in potato by increasing GA at this stage. *StGA2ox1* expression inhibits tuber development

The maintenance of an optimum cellular concentration of active CK is regulated by the balance between biosynthesis and catabolism. The first process of CK biosynthesis is catalyzed by *IPT* [129]. This enzyme catalyzes the transfer of an isopentenyl group from dimethylallyl disphosphate to an adenine nucleotide. An additional stage in the production of bioactive CK is the elimination of a ribose 5′-monophosphate group. The LONELY GUY (LOG) is a novel cytokinin-activating enzyme, directly converting inactive CK-ribotides to the free-base forms (Fig. 4).

Exogenous application of CK resulted in enhanced tuber development. In addition, overexpression of a CK biosynthesis gene, ipt, in potato resulted in more yield with reduced tuber weight.

Auxin, like CK, is involved in the process of cell expansion and enlargement. Auxin influences whole plant weight but yet stimulates tuberization, including both the number and weight of tubers [111]. Experimental surveys have indicated that auxin content and dynamics in the potato plant can influence the initiation, growth, and development of tubers [2]. Sergeeva et al. [130] inferred that the IAA movement from the shoot to the root and stolon is important for tuberization. The expression of the stolon-specific gene, *StYUC-like1*, *is* increased in stolon tips during tuberization. In addition, auxin production in stolon tips was confirmed by an analysis of auxin transport, showing a basic flux of auxin in stolon, transported from their apical meristems to stolons [3]. Early studies have suggested that a positive correlation between the auxin content and developmental rate was identified for tubers; however, auxin content was reduced with enhancing age and weight of tubers [131].

A comprehensive survey of the relationship between auxin content and cell division was conducted using young and mature tubers. The results showed that the auxin increased during the early stages of tuber formation, whereas the further developmental stage was expanded due to cell enlargement through auxin increase. At the same time, auxin content was increased twofold, whereas CK content was decreased twofold [131]. Another study showed that the process of tuber growth is normally related to an excessive presence of auxin content in the newly formed tubers [102].

Auxin transport and response are mediated through carrier proteins such as AUX1/LAX and PIN membrane-bound proteins in which the influx and efflux of auxins are controlled by these proteins, respectively [132]. Auxin directly conjugates to the SCF (TIR1) ligase, resulting in an increase in its affinity for the Aux/LAA proteins, a complex targeted for ubiquitin-mediated degradation. Under low- or high-auxin conditions, Aux/IAA inhibits AUXIN RESPONSE FACTORS (ARFs) and DNA-binding transcription factors. Aux/IAA degradation releases the ARFs which in turn may inhibit or facilitate auxin gene expression [99]. Auxin is regulated by potato receptor genes namely, *StTIR1a, StTIR1b*, and *StTIR1c*, as well as orthologs of the *AtTIR1* gene, *StAFB4* and *StAFB6*, and orthologs of *AtAFB* genes [133]. Maximum expression levels of this gene family were identified in leaves and stems and minimum levels were detected in roots [115]. Auxin transformation in leaves is strongly dependent on photoperiod, consequently transferred as the endogenous molecular and biochemical signaling cascades from shoots to stolon.

### Interaction between photoperiod and phytohormones controlling tuberization

The regulation of tuber formation is controlled by close interaction between the signaling transduction pathways of PHYB, GA, and miR172 in leaves and stolons. GA acts as a tuberization repressor, whereas StSP6A, miR172, and StBEL5 play roles as tuberization promoters under induced short-day conditions. In addition, POTH1 serves as a negative regulator of GA biosynthesis in the development of potato. It has been reported that POTH1 and StBEL5 enhanced tuberization under both short-day (SD) and long-day (LD) conditions in the stolon tip, through a balance between GA and CK to stimulate tuberization [106].

StBEL5 works in tandem with POTH1 transcription factor (TF). StSP6A protein and StBEL5 RNA are considered long-distance tuberization signals, moving through the phloem from the leaf to the stolon. In leaves, the transcription of StBEL5 is enhanced by light. PHYB controls the expression of StBEL5 through miR172 and RAP1, and of StFT through StCOL3 to regulate tuberization. The photoperiodic functional component in the leaf is merged with the regulation of GA biosynthesis and catabolic pathway implicated in the activation and inactivation of GAs at the stolon. It has been found that the reduction of GA in the stolon is required for tuberization initiation. An experimental survey revealed that auxin acts as a promoter of tuberization. Several auxin-related family genes, namely *StPIN* and *StARF*, are transcriptionally controlled during tuberization under short days. In addition, high levels of auxin extremely enhance tuber initiation and tuber development. GA stimulates stolon emergence in plants, whereas it has a repressive effect on tuber development. CK and auxin promote tuberization, reflecting a hormonal balance between GA, CK, and auxin during tuberization.

### Effects of multiple hormones on tuber maturation

After tuber maturation, potato tubers undergo a period of dormancy, where visible bud growth is inhibited [12]. The metabolism, physiology, and morphology of mature tubers are regulated by phytohormones namely ABA, GA, CK, and auxin. A previous experimental study has revealed that high doses of auxin treatment prolonged the dormancy state and inhibited sprout growth of potato tubers, whereas the low doses of auxin concentration stimulated sprout growth [12]. The utilization of auxin led to a significant increase in ethylene biosynthesis; thus, the auxin impact was indicated to be ethylene mediated [115]. Another study indicated that inhibition of tuber sprouting was related to auxin, without the ethylene mediation [12]. Further, an early experiment suggested that the high amounts of endogenous auxin in harvested tubers confirmed its relevance in tuber dormancy. At the onset of dormancy, measurement of the endogenous amount of auxin in tubers reached the maximum, gradually reducing afterwards until the initiation of sprouting [12–124].

A performed study in onion bulbs and *Gladiolus* cormels has shown that CK plays a key role in the termination of dormancy [134]. Besides CK and auxin, GA stimulated cell elongation during the vegetative growth of axial organs and delayed the growth of mature tubers. Experimental studies have revealed that GA acts as the tuberization inhibitor in potato. Koda and Okazawa [135] indicated that GA levels are high at tuber initiation, whereas GA levels are very low during tuber formation when the GA levels remained low to form mature tubers.

Utilization of growing tubers with GA decreased sucrose and starch content, suggesting that tuber formation and maturation were related to lower GA due to higher *StGA2ox1* expression. Overexpression of *StGA2ox1* led to a dwarf phenotype, decreased stolon growth, and earlier tuberization, whereas downregulation of *StGA2ox1* caused a reduction in expression levels of *StGA2ox1* leading to a normal plant phenotype, stolon swelling phenotype, and delayed tuberization. The proposed model suggested that *StGA2ox1* modified GA levels in the sub-apical stolon area at the initiation of tuberization. GA and ABA antagonistically mediate many growth and developmental processes, and their favorable balance is necessary for normal plant development. In potato, GA promotes stolon elongation and prevents tuber maturation. In contrast, ABA acts as a tuberization-stimulating component and its exogenous application accelerates tuberization in some potato cultivars. When ABA amount and ABA/GA ratio are enhanced, apical stolon development was delayed [136].

Experimental evidence has revealed that ABA stimulates tuberization by reciprocating the inhibitory impacts of GA. The presence of interplay between GA and ABA has revealed that the two signaling pathways are interconnected [137], suggesting that ABA inhibits the GA pathway through upregulation of ABA-responsive kinase PKABA1 (a member of the SnRK2 subfamily). The proposed hypothesis suggests that ABF transcription factors mediate crosstalk between ABA and GA signaling pathways.

**Fig. 5:**
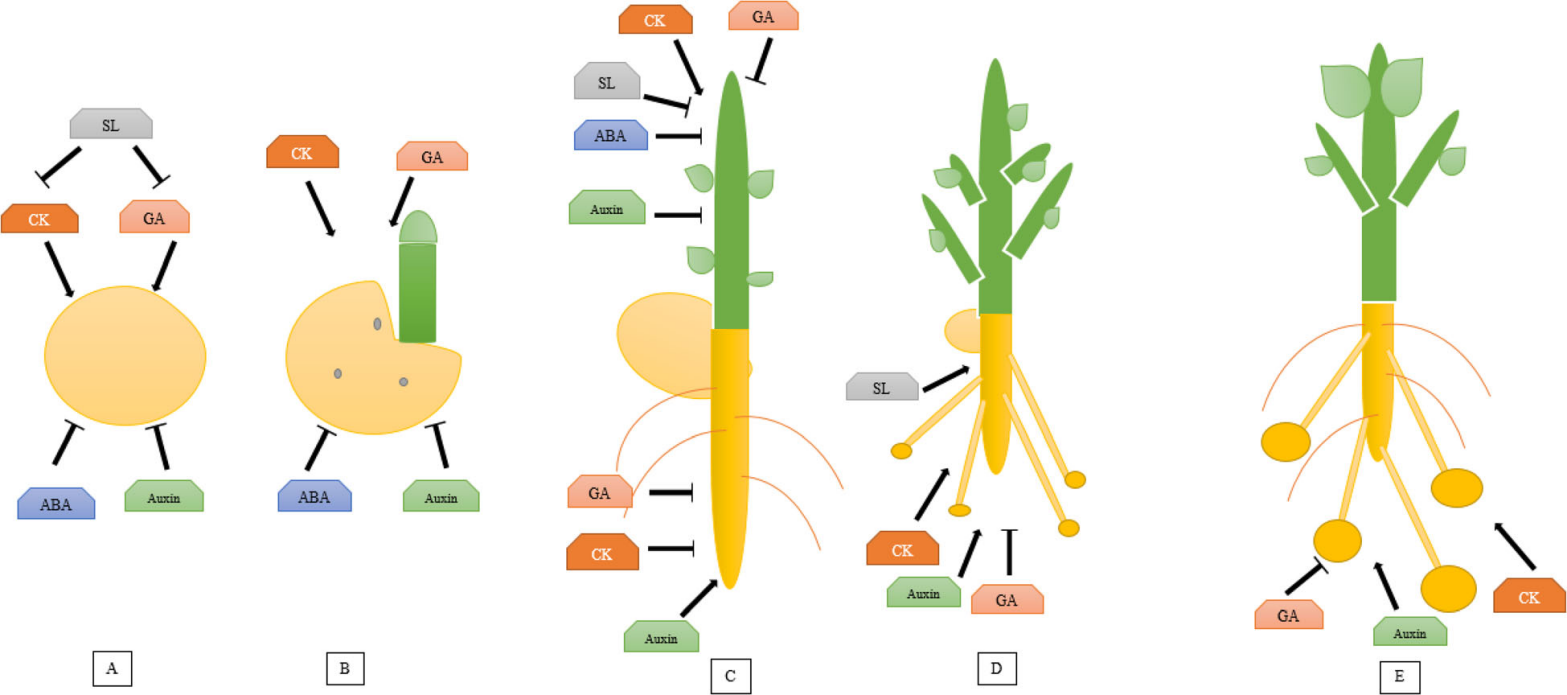
Several key regulators are involved during the tuber development, constituting a complex network. **A** ABA, auxin, SL maintained tuber dormancy. GA and CK stimulate tuber sprouting. **B** ABA and auxin are inhibitors to germinated tuber while, CK and GA are accelerators in sprouting. **C** Auxin acts as a stimulator and GA and CK act as a suppressor of root formation. CK enhanced the growth of shoot branching and bud growth, whereas ABA, auxin, GA, and SL are suppressors of shoot development. **D** At the tuber initiation stage, GA acts as an inhibitor, whereas auxin, CK, and SL stimulate tuberization. **E** During the tuber development stage, GA has an inhibitory role, whereas auxin and CK promote tuber development

In barley, the interplay of PKABA1 with HvABF1 and HvABF2 acts as a negative regulator of GA function [138]. In potato, StABF1 may regulate the ABA impacts on tuberization. It has been reported that phosphorylation of StABF1 is enhanced by ABA or tuber-forming conditions but is inhibited by GA. Consequently, ABF acts as a positive regulator of tuberization through interaction with ABA and GA. The ABF transgenic potato increased tuberization capacity, acting as a positive regulator and/or decreasing the effects of tuberization inhibitors. In potato, ABF4 increased tuberization through transcriptional deregulation of GA metabolism genes as well as enhanced high expression of *StGA20ox* and *StGA3ox* genes. However, regulation of tuber maturation is far less evaluated and studied than the hormonal control of tuber initiation. This is specifically evident from the small number of studies performed on this topic at the molecular and genetic levels (Fig. 5).

The accumulation of some substances is controlled by phytohormones. For example, ABA and auxin enhance the dormancy state whereas CK and GA accelerate sprouting. Many studies have shown that GA inhibited the accumulation of patatin, a marker for biochemical events related to the process of tuberization [139]. Application of exogenous GA in the tuber, stem, and whole plant causes the accumulation of major tuber proteins by GA inhibition [97].

## Conclusions

It is highlighted that several phytohormones are involved in the regulation of growth and development in potato.

GA and CK are actively increased during tuber sprouting, root growth, and tuber development, while auxin and ABA enhance tuber dormancy, shoot growth, and tuber initiation and development. Furthermore, the SL hormone interacts with other plant hormones either synergistically or antagonistically at tuber initiation and vegetative growth processes. A few potential candidates simplifying the interplay between auxin and GA and/or CK have been detected in the last several decades, but the detailed scientific base of molecular mechanisms remains ambiguous. It has been considered that the molecular characterization of the interplay between desired hormones during growth and development in potato requires to be undertaken. Although, there are aspects that have been explored extensively, such as the role of GA during the initiation of sprouting and the CK in tuberization, however, our insight of individual as well as combined roles of specific hormones during dormancy, tuber induction, and tuber development are very limited. It is necessary to find out how hormone networks behave and what type of changes endure during growth and development stages in potato. Also, hormonal links from sprouting to tuberization stage are well established. As a result, it is essential that the interconnection between hormonal network and reciprocal relationships with other genes of potato growth and development is investigated. We anticipate that future models on hormonal effects, individually and/or in combination, from tuber sprouting to tuber maturation provide a greater understanding of the intricate dynamics/mechanisms underlying the tightly synchronized biological processes.

## Data Availability

Not applicable.

## Abbreviations

CK: Cytokinin
GA: Gibberellin
ABA: Abscisic acid
SL: Strigolactone
LOG: LONELY GUY
ARF: Auxin responsive gene
CDK: Cyclin dependent kinase
CKX: CK oxidase/dehydrogenase
ABCB: ATP-binding cassette transporters
MAX: MORE AXILLARY BRANCHING
BRC1: BRANCHED1
*AXR*: *AUXIN RESISTANT*
SPY: SPINDLY
AHP6: *Arabidopsis* HISTIDINE PHOSPHOTRANSFER PROTEIN 6
IPT: *Isopentenyltransferase*
